# Effects of Prepartum Dietary Energy Level and Nicotinic Acid Supplementation on Immunological, Hematological and Biochemical Parameters of Periparturient Dairy Cows Differing in Parity

**DOI:** 10.3390/ani5030391

**Published:** 2015-09-08

**Authors:** Reka Tienken, Susanne Kersten, Jana Frahm, Liane Hüther, Ulrich Meyer, Korinna Huber, Jürgen Rehage, Sven Dänicke

**Affiliations:** 1 Institute of Animal Nutrition, Friedrich-Loeffler-Insitute (FLI), Federal Research Institute for Animal Health, Bundesallee 50, 38116 Brunswick, Germany; E-Mails: reka.tienken@fli.bund.de (R.T.); susanne.kersten@fli.bund.de (S.K.); liane.huether@fli.bund.de (L.H.); ulrich.meyer@fli.bund.de (U.M.); sven.daenicke@fli.bund.de (S.D.); 2 Department of Physiology, University of Veterinary Medicine Hanover, Bischofsholer Damm 15, 30173 Hanover, Germany; 3 Clinic for Cattle, University of Veterinary Medicine Hanover, Bischofsholer Damm 15, 30173 Hanover, Germany; E-Mail: juergen.rehage@tiho-hannover.de

**Keywords:** periparturient, niacin, dairy cow, immune system, PBMC, CD4, CD8

## Abstract

**Simple Summary:**

Several biological changes occur during the transition from late pregnancy to early lactation which is associated with a high susceptibility of health disorders. Nicotinic acid, as feed additive, is suggested to balance catabolic metabolism of periparturient dairy cows by attenuating lipolysis and impact production performance. This study provides information of the biological changes occurring around parturition with special emphasis on differences between primiparous and multiparous cows. Present results showed that energy-dense feeding *prepartum* did not result in metabolic imbalances *postpartum* in dairy cows which were similar in body condition score. Nicotinic acid supplementation did not reveal any effect.

**Abstract:**

The periparturient period is critical according to health, productivity and profitability. As this period is fundamental for the success of the lactation period, the interest in improving periparturient health by dietary supplements increased in recent years. The present study investigated the effects of feeding nicotinic acid (NA) combined with varying dietary energy densities on immunological, hematological and biochemical parameters of periparturient cows differing in parity. Thirty-six multiparous and 20 primiparous dairy cows were enrolled in the study 42 days before expected parturition date until 100 days *postpartum* with the half of the cows being supplemented with 24 g of NA/d. After parturition a diet with 30% concentrate was fed to all cows which was followed by different concentrate escalation strategies. Dietary NA supplementation was ceased on day 24 *postpartum*. Dietary NA increased (*P* = 0.010) serum nicotinamide concentrations (mean of 3.35 ± 1.65 µg/mL), whereas NA could not be detected. Present data emphasize that periparturient cows are faced with major physiological challenges and that both parity-groups have different prerequisites to adapt to those changes irrespective of NA supplementation. The overfeeding of energy to cows which were similar in body condition score had only minor effects on periparturient immune system function and the metabolism of those cows.

## 1. Introduction

A well-functioning immune system is a cornerstone for the protection against infectious and metabolic diseases. The periparturient period is associated with a high incidence of diseases due to profound changes in the hormonal, behavioral, digestive and immune system which impair immune function and lead to immunosuppression [1,2,3,4,5,6,7].

The reasons for the development of a periparturient immunosuppression are not fully known, but it seems to be a multifactorial process [3,7,8]. Indeed, early lactating dairy cows often develop a negative energy balance (NEB) due to a rapid increase in the requirements for milk production at a time when dry matter intake (DMI) is markedly depressed [9,10,11]. Dairy cows try to compensate this condition by lipid mobilization ensuring the availability of substrates for maintenance and milk production [9,10]. Lipid mobilization is associated with an increased release of non-esterified fatty acids (NEFA). In all, a massive release of NEFA can pave the way for hepatic lipidosis and ketosis due to an increased hepatic storage of fatty acids and an increased production of ß-hydroxybutyrate (BHB) [1,10]. It has recently been shown that changes in metabolism and hormone system, naturally occurring around parturition, have major impacts on the pattern and function of leukocytes [5,7,8,12,13,14]. Counts of leukocytes in the peripheral blood peak at calving, mainly as a consequence of increasing neutrophil counts [2,7,15,16], whereas the counts of lymphocytes decrease [2,8,13,16]. The proportions of lymphocyte subpopulations change also during this time [2,17]. Additionally, the responsiveness of peripheral blood mononuclear cells (PBMC) to mitogenic stimuli is markedly depressed and polymorphonuclear leukocytes (PMNL) show an impaired functionality during this period of production cycle [5,13]. 

Niacin serves as precursor for the coenzymes nicotinamide adenine dinucleotide (NAD^+^ and NADH) and nicotinamide adenine dinucleotide phosphate (NADP^+^ and NADPH) which are important participants in the regulation of energy metabolism, the maintenance of cellular redox status, the regulation of immunological functions, cell aging and cell death [18]. NAD^+^ is of great importance in the glycolytic pathway of peripheral T lymphocytes [19]. Further, it was shown that the dietary supplementation with nicotinic acid (NA) improves NAD^+^ content in human lymphocytes and reduces the vulnerability against oxygen-radical generated DNA damage [20]. Besides the expression of the niacin receptor, GPR109A, in diverse adipose tissues as well as the muscle, liver and brain tissue of bovines [21], this receptor is also expressed on the surface on mature neutrophil granulocytes [22], dendritic cells and peritoneal macrophages [23].

Feeding a diet high in concentrate proportion to late pregnant dairy cows pre-selected for a high body condition score (BCS), improved production performance, but lowered *postpartum* energy balance associated with increased NEFA concentrations [10]. The average incidence of subclinical ketosis (SCK) in postpartum dairy cows is 43% and those cows were more susceptible for health disorders postpartum [24]. Several authors observed negative effects of elevated NEFA and BHB concentrations on immunological function such as a reduced glucose consumption capacity in lymphocytes [25] and a reduced DNA synthesis and IgM secretion [14,26]. Further, the ability of PBMC to proliferate is negatively correlated to NEFA and BHB concentrations and it was shown that NEFA concentrations impair oxidative burst capacity [5]. 

As niacin is suggested to balance the catabolic situation *postpartum* by the down-regulation of lipolysis [27,28], it could be hypothesized that dietary niacin is able to attenuate negative consequences of *prepartum* nutrition and support cow’s health during periparturient period. 

Retrospectively, there has been no bovine study investigating the effects of dietary NA supplementation combined with different dry cow feeding strategies on the metabolism and the immune status of periparturient dairy cows differing in parity. Therefore, the present study aimed to offer new insights into the complex interactions between NA supplementation, dry cow nutrition and immunity during the periparturient period with special emphasis on a comparative observation of primiparous and multiparous cows under these conditions.

The objectives of the study were to investigate (1) the impacts of dry cow nutrition, (2) the effects of supplementing 24 g NA/d, and (3) the effects of parity on metabolism and immune system of periparturient dairy cows.

## 2. Materials and Methods 

### 2.1. Animals, Housing and Dietary Treatments 

In accordance with the German Animal Welfare Act, pertaining to the protection of experimental animals and approved by The Lower Saxony State Office for Consumer Protection and Food Safety (LAVES), Oldenburg, Germany, the trial was carried out at the experimental station of the Institute of Animal Nutrition, Friedrich-Loeffler-Institute (FLI), Brunswick, Germany. Fifty-six dairy cows (36 multiparous and 20 primiparous cows) of German Holstein breed were assigned to one of four dietary treatment groups. Aiming to provide homogeneous groups, multiparous cows were allocated by number of lactations (2.4 ± 0.1), body weight (698 ± 19 kg), BCS (3.1 ± 0.1) and the milk yield of previous lactation (6295 ± 234 kg, 200 d milk yield), whereas primiparous cows were distributed by body weight (589 ± 14 kg) and BCS (3.3 ± 0.1). 

The experiment started on day (d) 42 *prepartum* and was finished on 100 days in milk (DIM). Half of the cows received 24 g nicotinic acid (NA) per d (-NA) and cow, whereas the other half of the cows received no supplemental NA (-CON). The supplementation started on d −42 before expected parturition and was ceased on d +24. The NA supplement or the CON supplement was given as pelleted concentrate together with additional concentrate via the computerized concentrate feeding station (Insentec, B.V., Marknesse, The Netherlands). During *prepartum* period the cows received either a diet with low concentrate proportion (LC) (30% concentrate and 70% roughage mixture; 6.97 MJ NEL/kg dry matter (DM)) or a diet high in concentrate proportion (HC) (60% concentrate and 40% roughage mixture; 7.63 MJ NEL/kg DM). Cows were kept in a free stall barn which was split up in two group pens in accordance to the concentrate proportion of the *prepartum* diet. Twenty-six percent (LC group) or 56% (HC group) of the concentrate proportion were provided together with the roughage mixture as partial mixed ration, whereas the rest of the concentrate proportion (4%; including the NA or the CON supplement) was provided via the computerized concentrate feeding station (Insentec, B.V., Marknesse, The Netherlands). After parturition all cows were fed a diet consisting of 30% concentrate and 70% roughage mixture. The *postpartum* concentrate allowance increased up to 50% within 16 d in the LC groups and within 24 d in the HC groups. *Postpartum* dairy cows received 15% of the concentrate with the roughage mixture and 35% via the computerized concentrate feeding station (Insentec, B.V., Marknesse, The Netherlands). The roughage mixture of *prepartum* and *postpartum* period was composed of 50% corn silage and 50% grass silage on DM basis and was fed *ad libitum* via self-feeding stations (type RIC, Insentec B.V., Marknesse, The Netherlands). As the feed of the feeding troughs was provided for *ad libitum* intake, the available amount of concentrate and roughage mixture was adjusted for each cow twice weekly with regard to the current DMI of both components aiming to ensure the intended dietary forage to concentrate ratios of all dietary treatment groups. Cows had free access to water all the time. The composition of the dietary treatments is described in detail elsewhere [29].

The dietary treatment groups were: 1. Low concentrate diet without NA supplementation (LC-CON); 2. Low concentrate diet with NA supplementation (LC-NA); 3. High concentrate diet without NA supplementation (HC-CON); 4. High concentrate diet with NA supplementation (HC-NA). All diets were formulated according to the requirements given by the GfE [30].

###  2.2. Sample and Data Collection

Each cow was equipped with an ear transponder to record the daily individual feed and water intake.

For blood sampling cows were captured in a self-locking feed fence and blood was taken out of the *Vena jugularis externa* (07.30–09.00 h)*.* Samples of d −42 *prepartum* were taken before initiation of NA supplementation.

Samples for the determination of serum nicotinic acid (NA) and nicotinamide (NAM) concentrations were taken on d −42 (40 ± 6 d), −21 (17.7 ± 4.3 d), +14, +21, +28, +35 relative to parturition, whereas the blood samples for biochemical and hematological analyses and T lymphocyte phenotyping were taken on d −42 (40 ± 6 d), −21 (20.5 ± 2.1 d), −14 (13.6 ± 2.0 d), −7 (7.4 ± 1.7 d), −3 (2.7 ± 1.1 d), +3, +7, +14, +21, +28, +35, +42, +63, +84, +100 relative to parturition by using serum tubes for the analysis of biochemical variables and EDTA tubes for hematological analyses and T lymphocyte phenotyping. On d −42 (40 ± 6 d), −14 (11.6 ± 4.5 d), +3, +7, +14, +28, +42 and +100 blood samples for the isolation and proliferation assay of PBMC were obtained using heparinized vacutainer tubes.

### 2.3. Laboratory Methods

#### 2.3.1. Determination of Blood Niacin Concentration

The determination of NAM and NA was done using high-performance liquid chromatography (HPLC). Samples were thawed and carefully homogenized followed by protein precipitation and lipid extraction using ice cold ethanol and *n*-hexane, respectively. After centrifugation (20,800 *g* for 30 min) the supernatant was transferred into an amber flask and evaporated in a nitrogen stream at 40 °C. The residue was dissolved in aqueous mobile phase A. After filtration (amcro filter, PVDF, 0.45 µm) 20 μL were injected automatically into a HPLC system (Shimadzu, Kyoto, Japan). Samples were run through a C18 column (Inertsil ODS, 150 × 3 mm, 5 μm particle size, 150 Å pore size), using a binary gradient system. The mobile phase A consisted of 10 mM IPCC6 in ultrapure water at a pH of 2.3, mobile phase B was 100% acetonitrile. Quantification of NAM and NA were performed simultaneously by a multi wavelength detector at a wavelength of 260 nm. 

#### 2.3.2. Hematological and Biochemical Analyses

For the analyses of immunological and hematological values, the whole blood was analyzed immediately after blood sampling by using an automatic analyzer (Celltac αbMEK-6450, Nihon Kohden, Tokyo, Japan). The white blood cell profile included: the count of total leukocytes, lymphocytes and granulocytes. The measurement of granulocytes included basophil and neutrophil granulocytes. The red blood cell profile included: the count of red blood cells (RBC) with the investigation of hematocrit and hemoglobin and the red blood cell indices like mean corpuscular volume (MCV), mean corpuscular hemoglobin (MCH) and mean corpuscular hemoglobin concentration (MCHC). 

For serum preparation, the blood was incubated 30 minutes by 30 °C and then centrifuged at 2000× *g* for 15 minutes at 15 °C. Blood serum was stored at −80 °C until analysis. Concentrations of albumin, total cholesterol, triglycerides, urea and total protein as well as the activities of gamma glutamyl transferase (ɣ-GT), glutamate dehydrogenase and glutamic oxaloacetic transaminase (GOT) were determined spectrometrically by using the Eurolyser CCA 180 Vet system (Eurolyser Diagnostica GmbH, Salzburg, Austria). 

#### 2.3.3. Flow Cytometric Analysis

Phenotyping were performed by incubating whole blood samples with monoclonal fluorescein isothiocyanate (FITC)-labeled mouse anti bovine CD4 and phylloerythrin(PE)-labeled mouse anti-bovine CD8 antibodies or their respective isotype controls (AbD Serotec, Oxford, United Kingdom). Following 30 minutes of incubation in the dark, red blood cells were lysed and lymphocytes were simultaneously fixed by addition of FACS lysing solution (BD Bioscience, San Jose, CA, USA). Samples were analyzed on a FACS Canto II with High Througput Sampler using FACS DIVA software (BD Bioscience, San Jose, CA, USA). The spillover of both fluorochromes (FITC and PE) was automatically compensated by the software. For analyses the lymphoid population was gated based on its side- and forward-scattering properties. At least 10,000 lymphocytes were measured and relative counts of CD4^+^ and CD8^+^ positive cells were quantified. 

#### 2.3.4. Isolation and Culture of Peripheral Blood Mononuclear Cells

The isolation and preparation of PBMC was done according to the methodology by Renner *et al.* [31]. Isolated PBMC were kept frozen at −80 °C until further investigations. 

To evaluate the cell viability and Concanavalin A (Con A)-stimulated proliferation of PBMC, the Alamar Blue (AB) assay was used. This assay based on the reduction of the nonfluorescent resazurin to the fluorescent molecule, resofurin, by viable and metabolically active cells [32]. 

PBMC were thawed and washed firstly with Roswell Park Memorial Institute (RPMI)-1640 medium (Biochrom AG, Berlin, Germany) which was enriched with 5% fetal bovine serum (FBS, Biochrom AG, Berlin, Germany), 1 M HEPES (4-(2-hydroxyethyl)-1-piperazinethanesulfonic acid) buffer (Biochrom AG, Berlin, Germany), 2 mM L-glutamine (Biochrom AG, Berlin, Germany), 5 mM ß-mercaptoethanol (Biochrom AG, Berlin, Germany), 100 U/mL penicillin and 0.1 mg/mL streptomycin solution (Biochrom AG, Berlin, Germany). After centrifugation at 250× *g* for 8 minutes at room temperature, supernatants were removed and the pellet was suspended in the enriched RPMI-1640 medium. The adjustment of the cells to 1 × 10^6^ cells/mL was done using the trypan blue exclusion technique and a Neubauer counting chamber. Cell suspensions were assayed quintuplicate and were seeded into 96-well plates (1 × 10^5^ cells/well) and Con A (2.5 µg/mL final, Sigma-Aldrich, Germany) or RPMI-1640 medium were added up to a final volume of 200 µL/well. Following the procedures of Goyarts *et al.* [33], plates were then incubated for 72 h at 37 °C and 5% CO_2_ with subsequent centrifugation at 200× *g* for 5 minutes. 100 µL of the supernatant of each well were removed and 11 µL AB (AbD Serotec, Oxford, UK) were added to each well aiming to reach a dilution ratio of 1:10 which was followed by an incubation period of 2.5 h. The fluorescence of resorufin, the reaction product of the AB assay, was measured at 540 nm (excitation) and 590 nm (emission). 

###  2.4. Calculations and Statistics

The stimulation index (SI) was calculated using the following equation:

(1) $$\text{SI=}\frac{\text{Fluorescence of Con A}-\text{stimulated PBMC}}{\text{Fluorescence of unstimulated PBMC}}$$

The ratio of CD4^+^/CD8^+^ was calculated using following equation:

(2) $$\text{Ratio CD}4^{+}\text{/ CD}8^{+}\text{=}\frac{\text{Percentage of CD}4^{+}}{\text{Percentage of CD}8^{+}}$$

For statistical analysis measurements of daily water intake were condensed to weekly means. 

The PROC “MIXED” procedure of the SAS software package (SAS 9.4, SAS Inst. Inc., Cary, NC, USA) was used to determine the effects of the fixed effects on investigated variables. The fixed effects included dietary concentrate proportion (LC diet or HC diet), supplementation (CON or NA), parity (primiparous cows or multiparous cows), period (1st period: d 42 until birth; 2nd period: 1 until 28 DIM; 3rd period: 29 until 100 DIM) and the 4-way interaction between those factors. The random effect was cow within diet and period. The frequent measurements during experiment for each individual cow were considered as repeated measurement. After testing various covariance structures, a repeated-measures analysis using the compound symmetry was used, because this showed the lowest Akaike information criterion (AICC). Differences were considered to be significant at *P* < 0.05 and a tendency was noted if *P* < 0.10 and *P* > 0.05. The Tables are organized to represent LSMeans of the 4-way interaction (concentrate proportion × supplementation × parity × period), whereas the Figures represent mean values.

**Table 1:** The effects of different nutritional levels *prepartum* and nicotinic acid supplementation (24 g/d) on serum nicotinamide (NAM) concentrations of primiparous and multiparous cows during late gestation, periparturient period and early lactation (LSMeans ± SE).

Item		Diet					*P*-Value				
	P ^1^	LC-CON ^2^	LC-NA ^3^	HC-CON ^4^	HC-NA ^5^		C ^6^	S ^7^	P ^1^	T ^8^	C*S*P*T
NAM, µg/mL							0.738	0.010	0.538	<0.001	0.120
−42 until B ^9^	1	1.39 ± 0.49	3.25 ± 0.55	2.31 ± 0.58	2.20 ± 0.49						
	>1	2.08 ± 0.41	2.38 ± 0.45	1.86 ± 0.36	2.73 ± 0.41						
1–28 DIM	1	1.85 ± 0.46	4.40 ± 0.51	1.71 ± 0.51	2.79 ± 0.46						
	>1	2.37 ± 0.39	2.60 ± 0.42	2.45 ± 0.34	3.63 ± 0.39						
29–100 DIM	1	1.31 ± 0.57	2.32 ± 0.63	1.41 ± 0.63	2.01 ± 0.57						
	>1	2.54 ± 0.51	2.09 ± 0.52	2.15 ± 0.42	2.17 ± 0.48						

^1^ Parity; 1 = primiparous cows (LC-CON: n = 5; LC-NA: n = 4; HC-CON: n = 4; HC-NA: n = 5) and >1 = multiparous cows (LC-CON: n = 7; LC-NA: n = 6; HC-CON: n = 9; HC-NA: n = 7); ^2^ Low concentrate diet plus control concentrate (42 d *prepartum* until 24 DIM). Concentrate to roughage ratio 30:70 *prepartum*. After parturition the concentrate allowance increased from 30% to 50% within 16 d; ^3^ Low concentrate diet plus 24 g nicotinic acid/d (42 d *prepartum* until 24 DIM). Concentrate to roughage ratio 30:70 *prepartum*. After parturition the concentrate allowance increased from 30% to 50% within 16 d; ^4^ High concentrate diet plus control concentrate (42 d *prepartum* until 24 DIM). Concentrate to roughage ratio 60:40 *prepartum*. After parturition the concentrate allowance increased from 30% to 50% within 24 d; ^5^ High concentrate diet plus 24 g nicotinic acid/d (42 d *prepartum* until 24 DIM). Concentrate to roughage ratio 60:40 *prepartum*. After parturition the concentrate allowance increased from 30% to 50% within 24 d; ^6^ Concentrate proportion. Low concentrate diet (30% concentrate) or high concentrate diet (60% concentrate) either associated with a time-dependent increase of concentrate proportion up to 50% *postpartum*; ^7^ Supplementation. Concentrate premix containing nicotinic acid (24 g of NA/d and cow) or control concentrate (0 g of NA/d and cow); ^8^ Period. 1st period: d −42 until parturition; 2nd period: 1 until 28 DIM; 3rd period: 29 until 100 DIM; ^9^ The day of birth.

## 3. Results 

### 3.1. Animals 

Due to health problems, 9 cows were excluded from the experiment. Health problems included left abomasal displacement (two animals), mastitis (five animals), uterine rupture (one animal) and an accident (one animal). A total of 47 cows were considered for statistical analysis with following distribution: LC-CON: n = 5 primiparous and 7 multiparous cows; LC-NA: n = 4 primiparous and 6 multiparous cows; HC-CON: n= 4 primiparous and 9 multiparous cows; HC-NA: n = 5 primiparous and 7 multiparous cows.

### 3.2. Serum Niacin Concentration

Only NAM concentrations were detected in the present blood serum. NA supplementation increased serum NAM concentrations (Table 1). On d −42, mean NAM concentrations of NA supplemented cows were 2.11 ± 1.02 µg/mL and those of CON cows were 1.88 ± 0.90 µg/mL and did not differ statistically. During the time of supplementation mean NAM concentrations were 3.35 ± 1.65 µg/mL, whereas mean NAM concentrations of CON supplemented cows were 2.01 ± 1.10 µg/mL (data not shown). 

### 3.3. Biochemistry

With exception of serum urea concentrations, all investigated biochemical parameters were influenced by period (Supplementary material, Tables S1 and S2). NA supplementation increased GOT activity and tended to increase ɣ-GT activity (Supplementary material, Table S1). HC groups had higher GOT activities compared with LC groups (Supplementary material, Table S1). Multiparous cows had higher ɣ-GT activities as well as total protein, urea and cholesterol concentrations compared with primiparous cows (Supplementary material, Tables S1 and S2).

The interaction between dietary concentrate proportion, supplementation, parity and period influenced the activity of ɣ-GT and the urea concentrations differently (Supplementary material, Tables S1 and S2).

### 3.4. Hematology and Water Intake

With exception of primiparous cows receiving the LC diet, the counts of granulocytes peaked during the last week of gestation. A second increase was detected for multiparous cows on d +14 or +21, respectively. Counts of granulocytes were nearly stable during the 3rd period (data not shown). The counts of total leukocytes, granulocytes and lymphocytes were higher in primiparous than in multiparous cows (Table 2). 

Variables of the red blood cell profile and the water intake were influenced by period (Table 3). Primiparous cows had higher counts of RBC, whereas multiparous cows had higher MCV, MCH and water intake (Table 3). The interaction between dietary concentrate proportion, supplementation, parity and period influenced the water intake and MCHC as well as the hematocrit differently (Table 3).

**Table 2:** The effects of different nutritional levels *prepartum* and nicotinic acid supplementation (24 g/d) on counts of total leukocytes, granulocytes and lymphocytes of primiparous and multiparous cows during late gestation, periparturient period and early lactation (LSMeans ± SE).

Item		Diet					*P*-Value				
	P ^1^	LC-CON ^2^	LC-NA ^3^	HC-CON ^4^	HC-NA ^5^		C ^6^	S ^7^	P ^1^	T ^8^	C*S*P*T
Leukocytes, 10^3^/µL							0.298	0.341	0.001	0.172	0.327
−42 until B ^9^	1	9.6 ± 0.9	10.5 ± 1.0	8.8 ± 1.1	9.5 ± 0.9						
	>1	6.9 ± 0.8	8.1 ± 0.9	7.2 ± 0.7	9.0 ± 0.8						
1–28 DIM	1	10.3 ± 0.9	11.8 ± 1.0	8.9 ± 1.0	8.1 ± 0.9						
	>1	7.3 ± 0.8	8.4 ± 0.8	8.2 ± 0.7	7.8 ± 0.8						
29–100 DIM	1	8.8 ± 0.9	9.4 ± 1.0	9.0 ± 1.0	9.3 ± 0.9						
	>1	8.0 ± 0.8	7.2 ± 0.8	7.4 ± 0.7	7.0 ± 0.8						
Granulocytes, 10^3^/µL							0.347	0.238	0.050	0.007	0.060
−42 until B ^9^	1	4.7 ± 0.6	5.4 ± 0.6	4.4 ± 0.7	5.3 ± 0.6						
	>1	3.9 ± 0.5	4.3 ± 0.5	4.3 ± 0.4	5.3 ± 0.5						
1–28 DIM	1	5.6 ± 0.6	6.3 ± 0.6	4.1 ± 0.6	4.0 ± 0.6						
	>1	4.0 ± 0.5	4.2 ± 0.5	4.2 ± 0.4	4.1 ± 0.5						
29–100 DIM	1	4.0 ± 0.6	4.7 ± 0.6	3.8 ± 0.6	4.8 ± 0.6						
	>1	4.6 ± 0.5	3.5 ± 0.5	3.7 ± 0.4	3.8 ± 0.5						
Lymphocytes, 10^3^/µL							0.540	0.705	<0.001	0.529	0.713
−42 until B ^9^	1	4.1 ± 0.4	4.3 ± 0.4	3.9 ± 0.5	3.6 ± 0.4						
	>1	2.6 ± 0.3	3.0 ± 0.4	2.6 ± 0.3	3.2 ± 0.3						
1–28 DIM	1	4.1 ± 0.4	4.8 ± 0.4	3.9 ± 0.4	3.4 ± 0.4						
	>1	2.6 ± 0.3	3.2 ± 0.4	3.2 ± 0.3	3.2 ± 0.3						
29–100 DIM	1	4.1 ± 0.4	4.1 ± 0.4	4.3 ± 0.4	3.8 ± 0.4						
	>1	2.9 ± 0.3	3.0 ± 0.4	3.2 ± 0.3	2.8 ± 0.3						

^1^ Parity; 1 = primiparous cows (LC-CON: n = 5; LC-NA: n = 4; HC-CON: n = 4; HC-NA: n = 5) and >1 = multiparous cows (LC-CON: n = 7; LC-NA: n = 6; HC-CON: n = 9; HC-NA: n = 7); ^2^ Low concentrate diet plus control concentrate (42 d *prepartum* until 24 DIM). Concentrate to roughage ratio 30:70 *prepartum*. After parturition the concentrate allowance increased from 30% to 50% within 16 d; ^3^ Low concentrate diet plus 24 g nicotinic acid/d (42 d *prepartum* until 24 DIM). Concentrate to roughage ratio 30:70 *prepartum*. After parturition the concentrate allowance increased from 30% to 50% within 16 d; ^4^High concentrate diet plus control concentrate (42 d *prepartum* until 24 DIM). Concentrate to roughage ratio 60:40 *prepartum*. After parturition the concentrate allowance increased from 30% to 50% within 24 d; ^5^ High concentrate diet plus 24 g nicotinic acid/d (42 d *prepartum* until 24 DIM). Concentrate to roughage ratio 60:40 *prepartum*. After parturition the concentrate allowance increased from 30% to 50% within 24 d; ^6^ Concentrate proportion. Low concentrate diet (30% concentrate) or high concentrate diet (60% concentrate) either associated with a time-dependent increase of concentrate proportion up to 50% *postpartum*; ^7^ Supplementation. Concentrate premix containing nicotinic acid (24 g of NA/d and cow) or control concentrate (0 g of NA/d and cow); ^8^ Period. 1st period: d −42 until parturition; 2nd period: 1 until 28 DIM; 3rd period: 29 until 100 DIM; ^9^ The day of birth.

**Table 3:** The effects of different nutritional levels *prepartum* and nicotinic acid supplementation (24 g/d) on the counts of red blood cells (RBC), mean corpuscular volume (MCV), mean corpuscular hemoglobin (MCH), mean corpuscular hemoglobin concentration (MCHC), hemoglobin, hematocrit and water intake of primiparous and multiparous cows during late gestation, periparturient period and early lactation (LSMeans ± SE).

Item		Diet					*P*-Value				
	P ^1^	LC-CON ^2^	LC-NA ^3^	HC-CON ^4^	HC-NA ^5^		C ^6^	S ^7^	P ^1^	T ^8^	C*S*P*T
RBC, 10^3^/µL							0.216	0.519	<0.001	0.004	0.152
−42 until B^9^	1	6.1 ± 0.2	6.2 ± 0.2	5.8 ± 0.2	5.9 ± 0.2						
	>1	5.7 ± 0.2	5.5 ± 0.2	5.6 ± 0.2	5.6 ± 0.2						
1–28 DIM	1	6.2 ± 0.2	5.9 ± 0.2	5.7 ± 0.2	5.7 ± 0.2						
	>1	5.7 ± 0.2	5.4 ± 0.2	5.5± 0.2	5.4 ± 0.2						
29–100 DIM	1	6.3 ± 0.2	5.9 ± 0.2	5.7 ± 0.2	6.0 ± 0.2						
	>1	5.4 ± 0.12	5.1 ± 0.2	5.3 ± 0.2	5.4 ± 0.2						
MCV, fL							0.623	0.954	<0.001	<0.001	0.640
−42 until B ^9^	1	54.4 ± 1.7	56.0 ± 1.9	54.9 ± 1.9	53.4 ± 1.7						
	>1	58.9 ± 1.4	58.8 ± 1.5	60.5 ± 1.3	61.2 ± 1.4						
1–28 DIM	1	53.8 ± 1.7	59.1 ± 1.9	55.2 ± 1.9	58.9 ± 1.7						
	>1	54.7 ± 1.4	60.6 ± 1.5	52.5 ± 1.3	61.0 ± 1.4						
29–100 DIM	1	49.8 ± 1.7	55.7 ± 1.9	52.7 ± 1.9	56.0 ± 1.7						
	>1	52.3 ± 1.4	57.5 ± 1.5	49.2 ± 1.3	58.0 ± 1.4						
MCH, pg							0.580	0.583	<0.001	<0.001	0.794
−42 until B ^9^	1	18.6 ± 0.7	20.9 ± 0.8	18.3 ± 0.8	19.8 ± 0.7						
	>1	18.9 ± 0.6	20.7 ± 0.6	17.9 ± 0.5	20.3 ± 0.6						
1–28 DIM	1	16.5 ± 0.7	18.2 ± 0.8	17.0 ± 0.8	18.3 ± 0.7						
	>1	17.2 ± 0.6	18.8 ± 0.6	16.4 ± 0.5	18.7 ± 0.6						
29–100 DIM	1	15.9 ± 0.7	17.3 ± 0.8	16.2 ± 0.8	17.4 ± 0.7						
	>1	16.2 ± 0.6	17.9 ± 0.6	15.3 ± 0.5	18.8 ± 0.6						
MCHC, g/dL							0.944	0.013	0.829	<0.001	0.059
−42 until B ^9^	1	34.3 ± 0.6	35.2 ± 0.6	32.7 ± 0.7	33.1 ± 0.6						
	>1	34.9 ± 0.5	33.8 ± 0.5	32.6 ± 0.4	32.5 ± 0.5						
1–28 DIM	1	30.8 ± 0.6	30.8 ± 0.6	30.9 ± 0.6	31.1 ± 0.5						
	>1	31.4 ± 0.5	31.1 ± 0.5	31.2 ± 0.4	30.8 ± 0.5						
29–100 DIM	1	31.8 ± 0.6	31.0 ± 0.6	30.9 ± 0.6	31.1 ± 0.6						
	>1	31.0 ± 0.5	31.1 ± 0.5	31.1 ± 0.4	32.3 ± 0.5						
Hemoglobin, g/dL							0.450	0.083	0.338	<0.001	0.162
−42 until B ^9^	1	11.4 ± 0.3	11.7 ± 0.4	11.3 ± 0.4	10.8 ± 0.3						
	>1	11.2 ± 0.3	11.4 ± 0.3	10.4 ± 0.2	11.3 ± 0.3						
1–28 DIM	1	10.3 ± 0.3	10.3 ± 0.3	10.0 ± 0.4	9.9 ± 0.3						
	>1	9.8 ± 0.3	10.3 ± 0.3	9.4 ± 0.2	10.1 ± 0.3						
29–100 DIM	1	9.9 ± 0.3	9.3 ± 0.4	9.6 ± 0.3	8.9 ± 0.3						
	>1	9.3 ± 0.3	9.5 ± 0.3	9.1 ± 0.2	10.1 ± 0.3						
Hematocrit, %							0.275	0.418	0.460	<0.001	0.034
−42 until B ^9^	1	33.3 ± 0.8	33.2 ± 0.9	34.8 ± 0.9	32.2 ± 0.8						
	>1	32.1 ± 0.7	33.5 ± 0.8	31.6 ± 0.6	34.3 ± 0.7						
1–28 DIM	1	33.5 ± 0.8	33.4 ± 0.9	32.3 ± 0.9	31.9 ± 0.8						
	>1	31.2 ± 0.7	33.2 ± 0.7	30.0 ± 0.6	33.0 ± 0.7						
29–100 DIM	1	31.2 ± 0.8	30.1 ± 0.9	31.2 ± 0.9	28.6 ± 0.8						
	>1	29.9 ± 0.7	30.3 ± 0.7	29.3 ± 0.6	31.0 ± 0.7						
Water intake, L/d												0.387	0.277	<0.001	<0.001	<0.001
−42 until B ^9^	1	34 ± 5	35 ± 6	42 ± 6	33 ± 5						
	>1	37 ± 4	40 ± 5	46 ± 4	48 ± 4						
1–28 DIM	1	47 ± 5	59 ± 5	51 ± 5	46 ± 5						
	>1	69 ± 4	78 ± 4	73 ± 4	75 ± 4						
29–100 DIM	1	59 ± 5	77 ± 5	67 ± 5	62 ± 5						
	>1	79 ± 4	84 ± 4	91 ± 4	95 ± 4						

^1^ Parity; 1 = primiparous cows (LC-CON: n = 5; LC-NA: n = 4; HC-CON: n = 4; HC-NA: n = 5) and >1 = multiparous cows (LC-CON: n = 7; LC-NA: n = 6; HC-CON: n = 9; HC-NA: n = 7); ^2^ Low concentrate diet plus control concentrate (42 d *prepartum* until 24 DIM). Concentrate to roughage ratio 30:70 *prepartum*. After parturition the concentrate allowance increased from 30% to 50% within 16 d; ^3^ Low concentrate diet plus 24 g nicotinic acid/d (42 d *prepartum* until 24 DIM). Concentrate to roughage ratio 30:70 *prepartum*. After parturition the concentrate allowance increased from 30% to 50% within 16 d; ^4^ High concentrate diet plus control concentrate (42 d *prepartum* until 24 DIM). Concentrate to roughage ratio 60:40 *prepartum*. After parturition the concentrate allowance increased from 30% to 50% within 24 d; ^5^ High concentrate diet plus 24 g nicotinic acid/d (42 d *prepartum* until 24 DIM). Concentrate to roughage ratio 60:40 *prepartum*. After parturition the concentrate allowance increased from 30% to 50% within 24 d; ^6^ Concentrate proportion. Low concentrate diet (30% concentrate) or high concentrate diet (60% concentrate) either associated with a time-dependent increase of concentrate proportion up to 50% *postpartum*; ^7^ Supplementation. Concentrate premix containing nicotinic acid (24 g of NA/d and cow) or control concentrate (0 g of NA/d and cow); ^8^ Period. 1st period: d −42 until parturition; 2nd period: 1 until 28 DIM; 3rd period: 29 until 100 DIM; ^9^ The day of birth.

### 3.5. Lymphocyte Subpopulations

**Figure 1:**
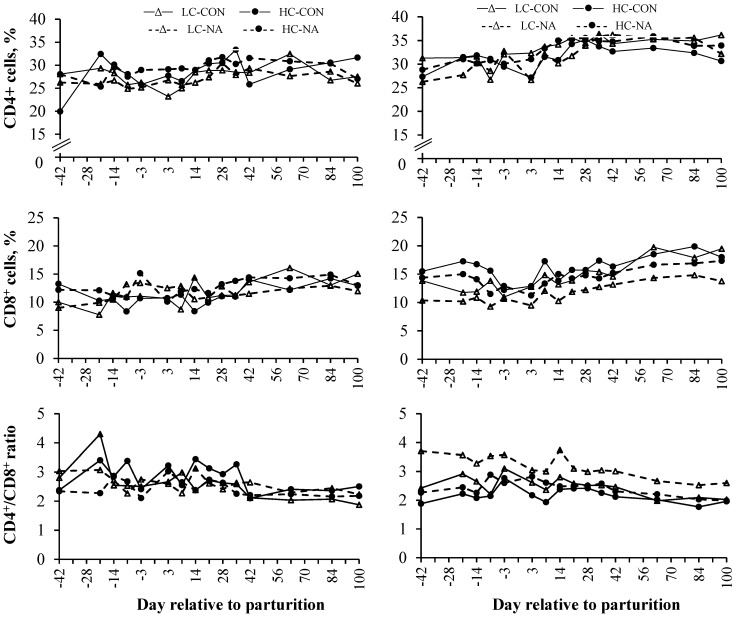
Mean proportions of CD8^+^ and CD4^+^ and the CD4^+^/CD8^+^ ratio in the whole blood of primiparous (left side) and multiparous cows (right side) during late gestation, periparturient period and early lactation.

Neither concentrate feeding nor NA supplementation influenced lymphocyte subpopulations in the present study (Table 4). All investigated parameters were influenced by period (Figure 1, Table 4). As shown in Figure 1, the CD8^+^ subpopulations decreased mainly during the last week of gestation and the first week of lactation with a dramatic decline on d −3 and +3 in multiparous cows. The CD8^+^ subpopulations increased continuously afterwards with a more pronounced increase in older cows. The CD4^+^ subpopulations showed lowest proportions around parturition which was followed by an increase until d +28 and a constant level during the last period. Multiparous cows had higher CD4^+^ and CD8^+^ subpopulations than primiparous cows (Figure 1, Table 4). The CD4^+^/CD8^+^ ratio of primiparous cows showed massive variations in the 1st and 2nd period, whereas those of multiparous cows showed a similar course between the four dietary treatment groups. The CD4^+^/CD8^+^ ratio of multiparous cows peaked firstly on d −7 or −3, respectively, and secondly on d +14. The CD4^+^/CD8^+^ ratio was lowest and constant in the 3rd period of the experiment in both parity-groups (Figure 1). 

**Table 4:** The effects of different nutritional levels *prepartum* and nicotinic acid supplementation (24 g/d) on CD4^+^ and CD8^+^ subpopulations and the ratio of CD4^+^/CD8^+^ of primiparous and multiparous cows during late gestation, periparturient period and early lactation (LSMeans ± SE).

Item		Diet					*P*-Value				
	P ^1^	LC-CON ^2^	LC-NA ^3^	HC-CON ^4^	HC-NA ^5^		C ^6^	S ^7^	P ^1^	T ^8^	C*S*P*T
CD4^+^/CD8^+^ ratio							0.335	0.528	0.718	<0.001	0.645
−42 until B ^9^	1	2.8 ± 0.3	2.8 ± 0.4	2.9 ± 0.4	2.5 ± 0.3						
	>1	2.5 ± 0.3	3.4 ± 0.3	2.3 ± 0.2	2.4 ± 0.3						
1–28 DIM	1	2.7 ± 0.3	2.5 ± 0.4	3.1 ± 0.4	2.7 ± 0.3						
	>1	2.6 ± 0.3	3.1 ± 0.3	2.3 ± 0.2	2.6 ± 0.3						
29–100 DIM	1	2.2 ± 0.3	2.5 ± 0.4	2.6 ± 0.4	2.2 ± 0.3						
	>1	2.2 ± 0.3	2.8 ± 0.3	2.0 ± 0.2	2.2 ± 0.3						
CD4^+^, %							0.368	0.955	<0.001	<0.001	0.128
−42 until B ^9^	1	27.0 ± 1.8	25.7 ± 2.0	27.8 ± 2.1	27.9 ± 1.8						
	>1	29.4 ± 1.5	29.2 ± 1.6	30.8 ± 1.3	30.2 ± 1.5						
1–28 DIM	1	26.9 ± 1.7	27.4 ± 2.0	28.8 ± 1.9	30.0 ± 1.7						
	>1	34.0 ± 1.5	31.0 ± 1.6	31.8 ± 1.3	34.0 ± 1.5						
29–100 DIM	1	28.5 ± 1.7	27.9 ± 1.9	30.8 ± 2.0	29.9 ± 1.8						
	>1	35.1 ± 1.5	35.0 ± 1.6	32.5 ± 1.3	34.6 ± 1.5						
CD8^+^, %							0.256	0.481	0.022	<0.001	0.797
−42 until B ^9^	1	10.4 ± 1.7	11.1 ± 1.8	11.0 ± 1.9	12.2 ± 1.6						
	>1	12.7 ± 1.4	10.6 ± 1.5	15.3 ± 1.2	13.9 ± 1.4						
1–28 DIM	1	11.2 ± 1.6	12.1 ± 1.8	10.3 ± 1.8	12.0 ± 1.6						
	>1	14.1 ± 1.4	11.4 ± 1.5	15.1 ± 1.2	13.7 ± 1.4						
29–100 DIM	1	13.6 ± 1.6	12.0 ± 1.8	12.8 ± 1.8	14.2 ± 1.6						
	>1	17.3 ± 1.4	13.8 ± 1.5	18.0 ± 1.2	16.0 ± 1.4						

^1^ Parity; 1 = primiparous cows (LC-CON: n = 5; LC-NA: n = 4; HC-CON: n = 4; HC-NA: n = 5) and >1 = multiparous cows (LC-CON: n = 7; LC-NA: n = 6; HC-CON: n = 9; HC-NA: n = 7); ^2^ Low concentrate diet plus control concentrate (42 d *prepartum* until 24 DIM). Concentrate to roughage ratio 30:70 *prepartum*. After parturition the concentrate allowance increased from 30% to 50% within 16 d; ^3^ Low concentrate diet plus 24 g nicotinic acid/d (42 d *prepartum* until 24 DIM). Concentrate to roughage ratio 30:70 *prepartum*. After parturition the concentrate allowance increased from 30% to 50% within 16 d; ^4^ High concentrate diet plus control concentrate (42 d *prepartum* until 24 DIM). Concentrate to roughage ratio 60:40 *prepartum*. After parturition the concentrate allowance increased from 30% to 50% within 24 d; ^5^ High concentrate diet plus 24 g nicotinic acid/d (42 d *prepartum* until 24 DIM). Concentrate to roughage ratio 60:40 *prepartum*. After parturition the concentrate allowance increased from 30% to 50% within 24 d; ^6^ Concentrate proportion. Low concentrate diet (30% concentrate) or high concentrate diet (60% concentrate) either associated with a time-dependent increase of concentrate proportion up to 50% *postpartum*; ^7^ Supplementation. Concentrate premix containing nicotinic acid (24 g of NA/d and cow) or control concentrate (0 g of NA/d and cow); ^8^ Period. 1st period: d −42 until parturition; 2nd period: 1 until 28 DIM; 3rd period: 29 until 100 DIM; ^9^ The day of birth.

### 3.6. PBMC Culture ex Vivo

**Figure 2:**
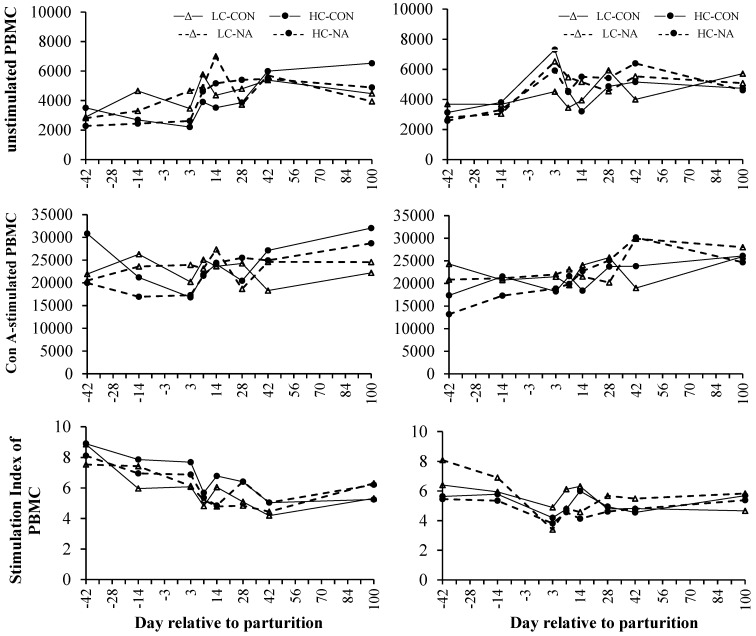
Mean viability of unstimulated, proliferation of Con A-stimulated and the stimulation index of peripheral blood mononuclear cells (PBMC) of primiparous (left side) and multiparous cows (right side) in Alamar blue assay.

Neither the dietary concentrate proportion nor the supplementation with NA influenced the viability of unstimulated PBMC, the proliferation of Con A-stimulated PBMC or the SI. However, those variables were influenced by period (Figure 2, Table 5). The basal viability of unstimulated PBMC and the proliferation of Con A-stimulated PBMC were similar on d −42 and −14 in both parity-groups. The viability of unstimulated PBMC peaked on d +3 in multiparous cows, whereas those of primiparous cows peaked on d +7 or +14, respectively. The viability of unstimulated PBMC in multiparous cows remained constant until the end of the experiment, whereas the viability of unstimulated PBMC in primiparous cows decreased from d +42 until +100, exceptional in group HC-NA. The proliferative capacity of Con A-stimulated PBMC in multiparous cows remained constant until d +7 followed by a slight increase until d +28. During the 3rd period the proliferation of Con A-stimulated PBMC remained nearly constant (Figure 2, Table 5). Present results indicate that the proliferative response of Con A-stimulated PBMC were constant until d +3 in primiparous cows. Primiparous Con A-stimulated PBMC peaked slightly during the first 2 weeks of lactation and increased from d +42 until +100. The viability of unstimulated and the proliferative response in Con A-stimulated PBMC was moderately lower *prepartum* compared with *postpartum* values. 

**Table 5:** The effects of different nutritional levels *prepartum* and nicotinic acid supplementation (24 g/d) on the viability of unstimulated and Con A-stimulated peripheral blood mononuclear cells (PBMC) and the stimulation index (SI) of primiparous and multiparous cows in Alamar blue assay (LSMeans ± SE).

		Diet					*P*-Value				
Item	P ^1^	LC-CON ^2^	LC-NA ^3^	HC-CON ^4^	HC-NA ^5^		C ^6^	S ^7^	P ^1^	T ^8^	C*S*P*T
Unstimulated PBMC, RFU							0.828	0.909	0.431	<0.001	0.895
−42 until B ^9^	1	3774 ± 806	3024 ± 960	3272 ± 1030	2358 ± 806						
	>1	3680 ± 681	2922 ± 736	3467 ± 601	2912 ± 706						
1–28 DIM	1	4567 ± 636	5076 ± 681	3377 ± 681	4454 ± 609						
	>1	4459 ± 515	5413 ± 566	5065 ± 465	5286 ± 516						
29–100 DIM	1	4932 ± 806	4825 ± 902	6263 ± 902	5183 ± 806						
	>1	4856 ± 681	5309 ± 736	4948 ± 601	5504 ± 681						
Stimulated PBMC ^10^, RFU							0.911	0.703	0.369	0.004	0.324
−42 until B ^9^	1	24,110 ± 3144	21,671 ± 3702	27,976 ± 3923	18,441 ± 3144						
	>1	22,526 ± 2657	20,987 ± 2870	19,442 ± 2344	14,816 ± 2735						
1–28 DIM	1	23,260 ± 2657	23,270 ± 2887	20,787 ± 2887	22,169 ± 2582						
	>1	22,721 ± 2182	21,567 ± 2385	20,535 ± 1955	21,602 ± 2188						
29–100 DIM	1	20,247 ± 3144	24,583 ± 3515	29,577 ± 3515	26,816 ± 3144						
	>1	22,474 ± 2657	28,960 ± 2870	24,955 ± 2344	27,417 ± 2657						
SI							0.997	0.846	0.007	<0.001	0.425
−42 until B ^9^	1	7.4 ± 0.7	7.4 ± 0.9	8.6 ± 0.9	7.5 ± 0.7						
	>1	6.2 ± 0.6	7.5 ± 0.7	5.7 ± 0.6	5.4 ± 0.6						
1–28 DIM	1	5.5 ± 0.6	5.2 ± 0.7	6.6 ± 0.7	5.9 ± 0.6						
	>1	5.5 ± 0.5	4.6 ± 0.6	4.9 ± 0.5	4.3 ± 0.5						
29–100 DIM	1	4.8 ± 0.7	5.4 ± 0.8	5.1 ± 0.8	5.6 ± 0.7						
	>1	4.7 ± 0.6	5.7 ± 0.7	5.1 ± 0.6	5.1 ± 0.6						

^1^ Parity; 1 = primiparous cows (LC-CON: n = 5; LC-NA: n = 4; HC-CON: n = 4; HC-NA: n = 5) and >1 = multiparous cows (LC-CON: n = 7; LC-NA: n = 6; HC-CON: n = 9; HC-NA: n = 7); ^2^ Low concentrate diet plus control concentrate (42 d *prepartum* until 24 DIM). Concentrate to roughage ratio 30:70 *prepartum*. After parturition the concentrate allowance increased from 30% to 50% within 16 d; ^3^ Low concentrate diet plus 24 g nicotinic acid/d (42 d *prepartum* until 24 DIM). Concentrate to roughage ratio 30:70 *prepartum*. After parturition the concentrate allowance increased from 30% to 50% within 16 d; ^4^ High concentrate diet plus control concentrate (42 d *prepartum* until 24 DIM). Concentrate to roughage ratio 60:40 *prepartum*. After parturition the concentrate allowance increased from 30% to 50% within 24 d; ^5^ High concentrate diet plus 24 g nicotinic acid/d (42 d *prepartum* until 24 DIM). Concentrate to roughage ratio 60:40 *prepartum*. After parturition the concentrate allowance increased from 30% to 50% within 24 d; ^6^ Concentrate proportion. Low concentrate diet (30% concentrate) or high concentrate diet (60% concentrate) either associated with a time-dependent increase of concentrate proportion up to 50% *postpartum*; ^7^ Supplementation. Concentrate premix containing nicotinic acid (24 g of NA/d and cow) or control concentrate (0 g of NA/d and cow); ^8^ Period. 1st period: d −42 until parturition; 2nd period: 1 until 28 DIM; 3rd period: 29 until 100 DIM; ^9^ The day of birth; ^10^ ConA-stimulated peripheral blood mononuclear cells (2.5 µg/mL).

A decrease in the SI was observed on d +3 in the case of multiparous cows. The first negative peak in SI of primiparous cows was observed on d +7 and was followed by a second negative peak on d +42. The SI of younger animals increased from d +42 until +100, whereas that of multiparous cows was constant during the 3rd period (Figure 2, Table 5). It was shown that the SI of primiparous cows was elevated *prepartum* compared with *postpartum* values, whereas those of multiparous cows did not differ distinctly pre- and *postpartum*. However, only the SI was influenced by parity resulting in a higher SI of primiparous cows compared with multiparous cows (Figure 2, Table 5).

## 4. Discussion 

The present study aimed to induce differences in the energy metabolism during *pre*- and *postpartum* period by feeding diets differing in concentrate proportion *prepartum,* different concentrate escalation strategies *postpartum* and the supplementation with NA. Data of performance parameters and energy metabolism including concentrations of NEFA, BHBA and glucose were reported previously [29]. In brief, HC groups consumed more DM, had higher energy intakes and a more positive calculated energy balance *prepartum*. However, NEFA and BHB concentrations as well as the BCS *prepartum* and at parturition were not affected. *Prepartum* dietary energy supply and NA supplementation had only minor effects on milk performance, and no effects on energy metabolism *postpartum* [29]. 

To our best knowledge, this is the first study investigating the effects of long-term NA supplementation on immunological, hematological and biochemical variables of periparturient dairy cows with special emphasis on primiparous and multiparous cows. Main findings of the present study were: (1) non-rumen protected NA increased serum NAM concentrations, however without affecting metabolism and immunological state, (2) feeding a diet high in energy density to dairy cows in late gestation had only a limited influence on the metabolism and the immunological state of periparturient dairy cows, (3) the periparturient period as well as the time of early lactation was associated with profound changes, and (4) primiparous and multiparous cows had different cellular prerequisites and capabilities to respond to onset of lactation.

###  4.1. Effects of Dietary Concentrate Level 

In an earlier study, the feeding of diets high in energy density *prepartum* resulted in a higher BCS at parturition which was associated with a higher loss in BCS and BW and a greater energy deficit *postpartum* [11]. The present experiment started 6 weeks before expected parturition and cows were grouped by their BCS to achieve homogenous groups with an average BCS of 3.1 ± 0.1 [29]. The present study with its selected feeding strategies however failed to affect the BCS, the energetic profile and the extent of *postpartum* lipolysis [29]*.* Contrarily, Schulz, *et al.* [10] conducted a similar feeding trial, but they grouped the animals by a higher and lower BCS; cows higher in BCS received a diet high in energy density (7.7 MJ NEL/kg DM) additionally. Those cows had an improved milk performance *postpartum* concomitant with elevated NEFA concentrations and higher GOT activities during the first two weeks of lactation [10]. The period of 6 weeks before parturition might be too short to provoke profound differences in the metabolic profile by dietary regimen when fed to cows similar in BCS. The absence of effects on production performance and energy metabolism might be one explanation for the lack of profound effects of the dietary concentrate proportion on investigated parameters of the present study. 

###  4.2. Effects of NA Supplementation

The dietary supplementation with 24 g NA/d elevated present serum NAM concentrations which reveals that an extra supply of NA and the use of non-rumen protected NA is able to boost physiologic NAM concentrations in the serum of periparturient dairy cows. 

The statements about NAM concentrations in times of NA supplementation in the literature are highly variable. Serum NAM concentrations were between 0.36 to 0.52 mg/L when supplementing 6 g non-rumen protected NA [34], 1.65 µg/mL [35] and 0.6 until 1.3 µg/mL [27] when supplementing 12 or 24 g rumen protected niacin/d, respectively. Those concentrations were quite lower, when converted to 24 g NA/d used in the present study. Niehoff, *et al.* [34] observed that serum NAM concentrations increased with increasing concentrate proportions, but present observations did not reveal an influence of the dietary concentrate proportion on serum NAM concentrations. 

Morey *et al.* [27] observed that 24 g rumen protected niacin/d suppressed plasma NEFA concentrations on 5 and 10 DIM. Those researchers detected both, NA and NAM, in the plasma using HPLC method. Contrary to this, NA was under the detection limit in the present study although using HPLC method. 

In an *in vitro* investigation, Kenez *et al.* [28] showed that in contrast to NAM, NA was a potent inhibitor of the lipolytic response in bovine adipose tissue via the GPR109A receptor-mediated pathway. Furthermore, it was stated that different immune cells express the nicotinic acid receptor, GPR109A, on its surface [22,23] and it was stated that a bright variety of biological effects are mediated via the binding of NA on this receptor [18]. Although the dietary NA supplementation elevated serum NAM concentrations, effects on biological, hematological and immunological parameters were almost missing in the present study. It could be hypothesized that the NA concentrations were too low to alter bovine metabolism and immune system and functionality, because serum NA concentrations were under the detection limit in the present study. Present results conceivably indicate that effects of dietary niacin are mainly driven by blood NA concentrations. Further, the results observed herein might provide evidence of a massive conversion of dietary NA to NAM.

###  4.3. Effects of Parity

It was stated that primiparous cows experience a higher “stress level” compared with multiparous cows. Gonzalez *et al.* [3] observed that primiparous cows experienced chronic stress during transition period and responded with higher cortisol concentrations after the administration of adrenocorticotropic hormone. Primiparous cows have a subordinated position when being integrated to the milking herd with far-reaching consequences for the welfare and health of younger animals [3]. As it was shown that glucocorticoids alter immune cell composition and function [3,7,36], it is worthwhile mentioning that parity-related differences in glucocorticoid levels imply differences in cellular perquisites and immune responses. Other researchers confirmed that variables of the red and white blood cell profile differ according to the age of dairy cows [4,8].

Furthermore, the ovarian steroid cycle differs between primiparous and multiparous cows which might account for parity-related difference in immune system. Tanaka *et al.* [37] observed that multiparous cows ovulated firstly 17.3 ± 6.3 d after calving, whereas first ovulation in primiparous cows was detected 31.8 ± 8.3 d after parturition. Differences in sexual cycle might explain the observation that multiparous cows of the present study experienced a second increase in the number of granulocytes on d +14 or +21 (data not shown) which is also observed by Schulz *et al.* [8]. 

As the MCV is calculated by dividing the hematocrit by the red blood cell count, and the MCH by dividing the hemoglobin concentration by the red blood cell count, it is clear that multiparous cows showed higher MCV and MCH compared with primiparous cows in this study. 

The blood of primiparous and multiparous cows of the present experiment contained different amounts of CD4^+^ and CD8^+^ subpopulations although both were in the same stage of production cycle. Shafer-Weaver *et al.* [6] observed that CD4^+^ subpopulations were forced to differentiate to T_H_-2 cells by cytokines which promotes humoral immunity by the secretion of IL-4, IL-5 and IL-10. They concluded that this, in part, skewing the immune system towards humoral immunity aiming to satisfy the requirements of antibody production for the newborn calf. As multiparous cows had higher numbers of T cell subpopulations and Mcgee *et al.* [38] observed that multiparous cows show a higher colostrum yield compared with primiparous cows, one reason for the parity related differences in cellular properties might be founded in the degree of antibody production for colostrum. Furthermore, these differences in cell-mediated immunity provide parity-related differences in the capability to respond to periparturient changes. Especially in multiparous cows, the proportions of CD8^+^ cells decreased on d −3 and +3, whereas the ratio of CD4^+^/CD8^+^ was elevated. Anderson, *et al.* [36] detected a transient decrease of the proportions of CD8^+^ cells and a transient increase of the proportions of CD4^+^ cells and the ratio of CD4^+^/CD8^+^ after glucocorticoid treatment. Together with our findings this may indicate that lymphocyte subpopulations of both parities behave differently to periparturient endocrine changes resulting in differences in the sensitivity and functional response. Furthermore, differences in the energetic profile may strengthen those differences as it is shown that blood metabolites like BHB and NEFA alter immune cells and their function [8,14]. Mehrzad and Zhao [39] determined that the CD4^+^/CD8^+^ ratio was above 4 in pluriparous cows and they stated that older cows suffer from a dysregulated immune status. However, this cannot be confirmed by the present results as the ratio of CD4^+^/CD8^+^ was not affected by parity. This suggests that exogenous and endogenous changes normally occurring around parturition have more influence on the immune status than aging and that both parities have their own mechanism to adapt to those changes. 

Based upon results, the PBMC of both parity-groups behaved differently in the periparturient period, which disagrees with observations of Renner, *et al.* [40]. The more pronounced decrease in SI in multiparous cows of the present study indicated that PBMC of primiparous cows had a higher ability to response to mitogens closely around parturition, although the basal PBMC viability of multiparous cows was higher at this time. Judging from the present results, this might be attributed to the more pronounced NEB at parturition accompanied by higher BHB concentrations in multiparous cows compared with primiparous cows [29]. Several authors showed that increased NEFA and BHB concentrations diminish responsiveness of immune cells [8,14]. The SI of primiparous cows showed a nadir on d +42 which almost agrees with observations of Renner, *et al.* [40] who detected lowest SI on d +49 after parturition. 

Despite sequential changes, the SI of primiparous cows showed a higher response capacity throughout the present experiment compared with multiparous cows. Although primiparous cows showed a lower energy balance *prepartum*, it was higher *postpartum* compared with that of multiparous cows [29] assuming that the more tensed metabolic situation around parturition of multiparous cows had long-lasting consequences on the immune function. Furthermore, glucose availability is necessary for an proper immune response [19] and as primiparous cows had higher glucose concentrations *prepartum* and slightly higher concentrations *postpartum* compared with multiparous cows in the present study [29], they might have better prerequisites for adequate response capacity. 

### 4.4. Effects of Time

Variables of the red blood cell profile [8,15,16] followed patterns also determined in previous studies investigating the periparturient period of dairy cows. 

The increase of granulocyte counts towards parturition was also observed previously [7,8,16] and might be due to an impaired trans-capillary migration capacity [12]. Glucocorticoids induce the down-regulation of the expression of adhesion molecules on neutrophils surface resulting in the de-margination and a reduced migration capability of neutrophils [12]. Furthermore, it was shown that glucocorticoids stimulate the release of circulating immature neutrophil granulocytes from bone marrow [12], in all, inducing neutrophilia. Results of Ster *et al.* [5] indicated that the oxidative burst of PMNL seemed to be less sensitive to NEFA and they stated that the high concentrations of pro-inflammatory cytokines naturally occurring around parturition may stimulate PMNL functions despite the presence of high NEFA concentrations. Thus an increase in granulocyte counts may support a sufficient capacity of immunological defense in the periparturient dairy cow. The periparturient period is associated with high cortisol concentrations which peak at parturition. This is due to several exogenous changes such as management and nutrition as well as endogenous changes such as the physiological adaption from late pregnancy to early lactation, both resulting in a higher “stress level” associated with the release of cortisol [6,7,9,36]. The use of the synthetic glucocorticoid dexamethasone influences the composition of T lymphocyte subpopulations and their functions [17,36]. In this sense, it is likely that a part of the observed changes may be explained by changes in cortisol concentrations naturally occurring around parturition. Observed proportions of CD4^+^ and CD8^+^ cells and their time-dependent changes were, to a large extent, congruent with previous observations [2,17]. 

Similar to observations of Meglia *et al.* [2] and Van Kampen and Mallard [17], the proportions of CD4^+^ and CD8^+^ were higher *postpartum* compared with *prepartum* proportions. Present results showed a decrease in the cell-mediated immunity in week −1 and + 1, because the percentage of CD4^+^ and CD8^+^ cells decreased and the results of the *ex vivo* experiments revealed a decreased functionality of T lymphocytes at this time as might be deduced from the decreased SI around parturition. This may indicate that the immune system of periparturient dairy cows is adjusted to humoral immunity which is confirmed by results of Shafer-Weaver *et al.* [6]. These researchers observed that CD4^+^ subpopulations are impelled to act as T_H_-2 cells rather than T_H_-1 cells at parturition; and they stated that endocrine changes further promote these modifications. The shift towards T_H_-2 cells may favor the susceptibility to diseases, because cytokines secreted by T_H_-2 cells may influence important cell-mediated defense mechanisms negatively [6]. 

Despite lower proportions of T lymphocytes in early lactation, T lymphocytes are more activated at this time [6]. However, present results cannot be used to make a statement about T cell differentiation and cytokine profile which needs to be clarified in further investigations. 

It has recently been shown that the ratio of CD4^+^/CD8^+^ provides information about the immune status and further predicts the risk of aging-related diseases and mortality [41]. In the bovine it was shown that a ratio higher than 2 indicates a proper immune status [42]. Present results indicate that the ratio of CD4^+^/CD8^+^ showed nearly stable values varying from 2 to 2.5 from d +42 until the end of the experiment. This observation provides evidence that a balanced metabolic situation influence immune status positively and that a CD4^+^/CD8^+^ ratio of 2 to 2.5 is a good threshold for indicating a proper immune status. However, Mehrzad and Zhao [39] suggested that a CD4^+^/CD8^+^ ratio above 4 displays immunological dysregulation which might be confirmed by the present observations of an elevated CD4^+^/CD8^+^ (above 2.5) closely around parturition. 

Results of the present study showed that the basal metabolic activity of PBMC was stimulated closely around parturition although the responsiveness of PBMC to mitogenic stimuli was impaired. This might be caused by changes in subpopulations and their cytokines as discussed before, but the exact reasons require further investigation. In a study conducted by Renner, *et al.* [13] the SI of primiparous cows was higher *prepartum* compared with the first day *postpartum.* This was also observed in the present study in both parity groups. Lacetera, *et al.* [14] stated that the functionality of PBMC represented by DNA synthesis, IgM secretion as well as IFN-ɣ production, was moderately depressed when NEFA concentrations exceed 0.5 mmol/L. Furthermore, results of Ster, *et al.* [5] and Lacetera, *et al.* [26] confirmed observations that the functionality of PBMCs depends on circulating NEFA concentrations. The present decrease in SI might also be a consequence of elevated NEFA concentrations as those concentrations were higher than 0.5 mmol/L in both parity-groups at this time [29]. The transition period is a multifactorial approach for dairy cows assuming that changes in the proliferation capacity and the functionality of other immune cells might also be a result of changes in bioenergetic demands of immune cells and fluctuating nutrient availability during the periparturient period.

The immunological response to an inflammatory stimulus induces shifts in cellular metabolism to satisfy increased nutrient requirements [19]. For example, the activation of T cells stimulates a switch from oxidative phosphorylation to enhanced glycolysis. To fulfill the high glucose demand, the expression of glucose transporter 1 is enhanced aiming to satisfy the requirements and providing carbon for biosynthetic precursors [19]. As shown above, glucose plays an important role in immune cell metabolism and the decrease in glucose concentration around parturition [29], may also be a factor influencing the SI of PBMC and other immune cells around parturition. Furthermore, Anderson *et al.* [36] observed that the proliferation of phytohaemagglutinin-stimulated PBMC was markedly depressed by dexamethasone treatment. Therefore, this may suggest that the high levels of cortisol around parturition [7] also influence the functionality of PBMC. It has recently been shown that the SI increases with progressed lactation [13] which seems to be the result of normalized NEFA and BHBA concentrations or increasing glucose concentrations reflecting a balanced metabolic status and a sufficient nutrient availability [29].

## 5. Conclusions 

An important message of this study is that feeding a diet high in concentrate proportion and energy-density for the last 6 weeks of pregnancy to dairy cows similar in BCS has only limited effects on the immunological and hematological system as well as the metabolism. Furthermore, the supplementation of non-rumen protected NA increases serum NAM concentrations, but has no major effects on investigated parameters.

Present results strengthen the considerations that the periparturient period is the most stressful period for dairy cows, because a lot of exogenous and endogenous changes occur which affect the immune system. However, on the basis of the present results a massive immunosuppression around parturition could not be confirmed. However, it has to be kept in mind that only a small part of the whole was investigated in the present study. In all, present results gain more insights in the biology of periparturient primiparous and multiparous cows. It is shown that cellular prerequisites and functional capacities depend massively on the age and the energy balance of dairy cows. There is still need for further research to understand the complex connection of endocrine system, metabolism, immune system and immune functionality in periparturient dairy cows. Additionally, the special focus on primiparous and multiparous cows is indispensable to establish appropriate measures aiming to increase periparturient health and productivity. 

## Supplementary Files

Supplementary File 1
