# Supplementary material for: Effects of Prepartum Dietary Energy Level and Nicotinic Acid Supplementation on Immunological, Hematological and Biochemical Parameters of Periparturient Dairy Cows Differing in Parity
Source: Animals (Basel). 2015 Sep 8;5(3):910–33. doi: 10.3390/ani5030391 (PMC4598713; doi:10.3390/ani5030391)
Supplement: Supplementary File 1 [file animals-05-00391-s001.pdf]

Supplementary

Effects of Prepartum Dietary Energy Level and Nicotinic Acid Supplementation on Immunological, Hematological and Biochemical Parameters of Periparturient Dairy Cows Differing in Parity

Table S1. The effects of different nutritional levels *prepartum* and nicotinic acid supplementation (24 g/d) on serum activities of glutamic-oxaloacetic-transaminase (GOT), gamma-glutamyl transferase (ɣ-GT) and glutamatedehydrogenase (GLDH) of primiparous and multiparous cows during late gestation, periparturient period and early lactation (LSMeans ± SE).

Item	P [1]	Diet				*P*-Value				
		LC-CON [2]	LC-NA [3]	HC-CON [4]	HC-NA [5]	C [6]	S [7]	P [1]	T [8]	C*S*P*T
GOT, U/L						0.032	0.022	0.726	<0.001	0.781
−42 until B [9]	1	58.4 ± 10.4	58.9 ± 11.5	51.8 ± 12.6	75.6 ± 10.4					
	>1	46.0 ± 8.9	71.8 ± 9.6	55.9 ± 7.7	72.0 ± 8.9					
1–28 DIM	1	77.5 ± 10.2	74.3 ± 11.4	70.3 ± 11.4	100.2 ±10.2					
	>1	67.3 ± 8.6	96.3 ± 9.3	90.8 ± 7.6	100.3 ± 8.6					
29–100 DIM	1	84.8 ± 10.3	80.0 ± 11.4	91.1 ± 11.4	100.8 ±10.2					
	>1	73.3 ± 8.6	80.1 ± 9.4	97.0 ± 7.6	93.3 ± 8.6					
ɣ-GT, U/L						0.384	0.061	0.002	<0.001	0.066
−42 until B [9]	1	16.2 ± 3.6	20.4 ± 4.0	16.2 ± 4.1	17.6 ± 3.6					
	>1	18.3 ± 3.0	22.2 ± 3.3	20.6 ± 2.7	24.1 ± 3.0					
1–28 DIM	1	18.7 ± 3.5	22.9 ± 3.9	20.6 ± 3.9	21.2 ± 3.5					
	>1	18.2 ± 3.0	30.9 ± 3.2	31.5 ± 2.6	27.3 ± 3.0					
29–100 DIM	1	20.0 ± 3.5	24.9 ± 3.9	25.0 ± 3.9	26.0 ± 3.5					
	>1	25.6 ± 3.0	40.6 ± 3.5	35.4 ± 2.6	34.6 ± 3.0					
GLDH, U/L						0.826	0.492	0.301	<0.001	0.867
−42 until B [9]	1	6.9 ± 8.4	9.9 ± 9.3	6.6 ± 9.7	7.7 ± 8.4					
	>1	7.4 ± 7.2	12.0 ± 7.8	11.0 ± 6.2	12.3 ± 7.2					
1–28 DIM	1	8.9 ± 8.3	25.2 ± 9.2	16.4 ± 9.3	12.8 ± 8.3					
	>1	17.2 ± 7.0	33.3 ± 7.5	25.2 ± 6.1	16.7 ± 6.9					
29–100 DIM	1	21.9 ± 8.3	40.3 ± 9.2	43.5 ± 9.2	38.3 ± 8.2					
	>1	37.7 ± 7.0	34.8 ± 7.5	43.1 ± 6.1	31.3 ± 6.9					

[1] Parity; 1 = primiparous cows (LC-CON: n = 5; LC-NA: n = 4; HC-CON: n = 4; HC-NA: n = 5) and >1 = multiparous cows (LC-CON: n = 7; LC-NA: n = 6; HC-CON: n = 9; HC-NA: n = 7); [2] Low concentrate diet plus control concentrate (42 d *prepartum* until 24 DIM). Concentrate to roughage ratio 30:70 *prepartum*. After parturition the concentrate allowance increased from 30% to 50% within 16 d; [3] Low concentrate diet plus 24 g nicotinic acid/d (42 d *prepartum* until 24 DIM). Concentrate to roughage ratio 30:70 *prepartum*. After parturition the concentrate allowance increased from 30% to 50% within 16 d; [4] High concentrate diet plus control concentrate (42 d *prepartum* until 24 DIM). Concentrate to roughage ratio 60:40 *prepartum*.

After parturition the concentrate allowance increased from 30% to 50% within 24 d; [5] High concentrate diet plus 24 g nicotinic acid/d (42 d *prepartum* until 24 DIM). Concentrate to roughage ratio 60:40 *prepartum*. After parturition the concentrate allowance increased from 30% to 50% within 24 d; [6] Concentrate proportion. Low concentrate diet (30% concentrate) or high concentrate diet (60% concentrate) either associated with a time-dependent increase of concentrate proportion up to 50% *postpartum*; [7] Supplementation. Concentrate premix containing nicotinic acid (24 g of NA/d and cow) or control concentrate (0 g of NA/d and cow); [8] Period. 1st period: d −42 until parturition; 2nd period: 1 until 28 DIM; 3rd period: 29 until 100 DIM; [9] The day of birth.

Table S2. The effects of different nutritional levels *prepartum* and nicotinic acid supplementation (24 g/d) on total protein (TP), albumin (ALB), urea, triglyceride (TAG) and cholesterol (CHOL) concentrations of primiparous and multiparous cows during late gestation, periparturient period and early lactation (LSMeans ± SE).

		Diet				*P*-Value				
**Item**	**P [1]**	**LC-CON [2]**	**LC-NA [3]**	**HC-CON [4]**	**HC-NA [5]**	**C [6]**	**S [7]**	**P [1]**	**T [8]**	**C*S*P*T**
TP, g/dL						0.742	0.801	<0.001	<0.001	0.001
−42 until B [9]	1	62.0 ± 1.9	65.6 ± 2.0	62.3 ± 2.1	61.2 ± 1.9					
	>1	70.8 ± 1.6	72.9 ± 1.7	71.4 ± 1.4	71.4 ± 1.6					
1–28 DIM	1	73.0 ± 1.8	69.1 ± 2.0	68.6 ± 2.0	69.6 ± 1.8					
	>1	71.1 ± 1.5	74.4 ± 1.7	78.4 ± 1.3	73.9 ± 1.5					
29–100 DIM	1	68.4 ± 1.8	71.8 ± 2.0	69.8 ± 2.0	70.1 ± 1.8					
	>1	75.1 ± 1.5	75.9 ± 1.7	75.6 ± 1.3	73.8 ± 1.5					
ALB, g/dL						0.516	0.176	0.102	0.036	0.054
−42 until B [9]	1	34.9 ± 0.8	37.6 ± 0.8	35.1 ± 0.9	34.8 ± 0.8					
	>1	34.9 ± 0.7	35.2 ± 0.7	36.9 ± 0.6	36.4 ± 0.7					
1–28 DIM	1	35.0 ± 0.7	35.2 ± 0.8	32.7 ± 0.8	35.0 ± 0.7					
	>1	35.0 ± 0.6	34.2 ± 0.7	36.3 ± 0.6	36.4 ± 0.6					
29–100 DIM	1	34.4 ± 0.8	36.4 ± 0.8	33.7 ± 0.8	35.7 ± 0.7					
	>1	35.2 ± 0.6	34.7 ± 0.7	37.0 ± 0.6	35.8 ± 0.6					
Urea, mg/dL						0.404	0.586	0.042	0.661	0.034
−42 until B [9]	1	26.9 ± 2.4	30.5 ± 2.7	29.1 ± 2.8	31.6 ± 2.4					
	>1	26.6 ± 2.1	34.4 ± 2.3	34.5 ± 1.8	38.0 ± 2.1					
1–28 DIM	1	31.7 ± 2.4	34.3 ± 2.7	29.8 ± 2.7	33.6 ± 2.4					
	>1	26.3 ± 2.0	33.3 ± 2.2	31.9 ± 1.8	28.7 ± 2.0					
29–100 DIM	1	27.9 ± 2.4	31.9 ± 2.7	30.3 ± 2.7	29.7 ± 2.4					
	>1	32.7 ± 2.0	32.6 ± 2.2	31.4 ± 1.8	27.8 ± 2.0					
TAG, mg/dL						0.527	0.670	0.467	<0.001	0.522
−42 until B [9]	1	22.6 ± 1.1	22.8 ± 1.2	20.7 ± 1.3	22.2 ± 1.1					
	>1	22.9 ± 0.9	24.1 ± 1.0	21.2 ± 0.8	21.2 ± 0.9					
1–28 DIM	1	10.2 ± 1.1	10.1 ± 1.2	9.7 ± 1.2	9.5 ± 1.1					
	>1	10.4 ± 0.9	9.6 ± 1.0	11.1 ± 0.8	11.1 ± 0.9					
29–100 DIM	1	10.4 ± 1.1	11.2 ± 1.2	10.8 ± 1.2	11.1 ± 1.1					
	>1	10.9 ± 0.9	10.5 ± 1.0	11.4 ± 0.8	11.7 ± 0.9					
CHOL,										

mg/dL										
−42 until B [9]	1	81.3 ± 10.2	93.0 ± 11.3	89.9 ± 11.5	106.2 ± 10.2	0.499	0.726	0.001	<0.001	0.095
	>1	77.0 ± 8.6	97.2 ± 9.4	77.5 ± 7.6	87.7 ± 8.7					
1–28 DIM	1	84.8 ± 10.0	99.8 ± 11.2	82.4 ± 11.2	97.8 ± 10.0					
	>1	85.5 ± 8.5	110.4 ± 9.2	77.9 ± 7.5	97.8 ± 8.5					
29–100 DIM	1	142.6 ± 10.1	169.0 ± 11.2	152.8 ± 11.2	171.6 ± 10.0					
	>1	134.8 ± 8.5	183.8 ± 9.2	133.4 ± 7.5	158.0 ± 8.5					

[1] Parity; 1 = primiparous cows (LC-CON: n = 5; LC-NA: n = 4; HC-CON: n = 4; HC-NA: n = 5) and >1 = multiparous cows (LC-CON: n = 7; LC-NA: n = 6; HC-CON: n = 9; HC-NA: n = 7); [2] Low concentrate diet plus control concentrate (42 d *prepartum* until 24 DIM). Concentrate to roughage ratio 30:70 *prepartum*. After parturition the concentrate allowance increased from 30% to 50% within 16 d; [3] Low concentrate diet plus 24 g nicotinic acid/d (42 d *prepartum* until 24 DIM). Concentrate to roughage ratio 30:70 *prepartum*. After parturition the concentrate allowance increased from 30% to 50% within 16 d; [4] High concentrate diet plus control concentrate (42 d *prepartum* until 24 DIM). Concentrate to roughage ratio 60:40 *prepartum*. After parturition the concentrate allowance increased from 30% to 50% within 24 d; [5] High concentrate diet plus 24 g nicotinic acid/d (42 d *prepartum* until 24 DIM). Concentrate to roughage ratio 60:40 *prepartum*. After parturition the concentrate allowance increased from 30% to 50% within 24 d; [6] Concentrate proportion. Low concentrate diet (30% concentrate) or high concentrate diet (60% concentrate) either associated with a time-dependent increase of concentrate proportion up to 50% *postpartum*; [7] Supplementation. Concentrate premix containing nicotinic acid (24 g of NA/d and cow) or control concentrate (0 g of NA/d and cow); [8] Period. 1st period: d −42 until parturition; 2nd period: 1 until 28 DIM; 3rd period: 29 until 100 DIM; [9] The day of birth.

© 2015 by the authors; licensee MDPI, Basel, Switzerland. This article is an open access article distributed under the terms and conditions of the Creative Commons Attribution license (http://creativecommons.org/licenses/by/4.0/).
